# An unusual cause of chronic neuropathic pain: report of a case of multiple intradural spinal arachnoid cysts and review of the literature

**DOI:** 10.1007/s00701-023-05732-1

**Published:** 2023-08-01

**Authors:** Victor Gabriel El-Hajj, Erik Edström, Adrian Elmi-Terander, Alexander Fletcher-Sandersjöö

**Affiliations:** 1 Department of Clinical Neuroscience, Karolinska Institutet, Stockholm, Sweden; 2 Capio Spine Center Stockholm, Löwenströmska Hospital, Box 2074, 194 02 Upplands Väsby, Sweden; 3 Department of Surgical Sciences, Uppsala University, Uppsala, Sweden

**Keywords:** Spinal arachnoid cysts, Chronic neuropathic pain, Back pain, Spine

## Abstract

Spinal arachnoid cysts (SACs) arise either intra- or extradurally and are usually solitary, while cases of multiple SACs have been scarcely reported in the literature. Herein, we report on a rare case of multiple and recurring intradural spinal arachnoid cysts (SACs) causing severe spinal cord compression and neuropathic radicular pain, in a 35-year-old female with a 10-year follow-up. Two separate attempts at surgery were performed but only provided temporary relief since the cysts recurred and new ones formed along the entirety of the spinal cord. Finally, a conservative approach with physiotherapy and a combination of analgesic medications was pursued.

**Supplementary Information:**

The online version contains supplementary material available at 10.1007/s00701-023-05732-1.

## Background

Spinal arachnoid cysts (SACs) are rare occurrences that have been classified as part of the larger group of meningeal pathologies called spinal meningeal cysts [3]. While spinal extradural arachnoid cysts (extradural SACs) are more common than spinal intradural arachnoid cysts (intradural SACs), both lesions are typically single, sporadic, and recur infrequently after treatment [8]. The recurrence rates after surgical excision of intradural SACs in the adult and pediatric populations have been estimated at 13% [1] and 9.5% [4], respectively. While a 2009 report identified about 20 cases of multiple extradural ones [2], the prevalence of multiple intradural SACs within the literature has yet to be covered.

In this work, we present a case of multiple primary, recurring, and de novo forming intradural SACs in an adult female, with the aim of describing the diagnosis, management, and outcome of this rare occurrence, with a 10-year follow-up. In addition, we reviewed the literature on similar cases and provide a summary of the current knowledge. This report is, to the best of our knowledge, the first to describe the long-term outcomes in a patient with multiple de novo forming and recurring intradural SACs.

## Case presentation

Written informed consent for both the creation and publishing of the case report with the associated images was provided by the patient in question. The CARE checklist is provided as supplementary (Supplementary file 1).

### Patient presentation

A 35-year-old female presented at her general practitioner’s office with a 3-year history of progressive neck and back pain. She also experienced weakness of the hands and frequently dropped items. Her previous medical history included surgery for a posterior fossa arachnoid cyst at the age of 14. On evaluation, she had radiating pain along the C7 and C8 dermatomes as well as a burning back pain along the level of the scapula, radiating anteriorly. A shooting back pain, just below the inferior angle of the scapula, was provoked by motion. An MRI examination was performed, and the patient was subsequently referred to the university hospital’s neurosurgical department (Table 1).

**Table 1:** History and timeline of the patient’s diagnosis, treatment, and follow-up

Time	Event	Description
− 22 y	Surgery in the posterior fossa	At 14 years old, the patient underwent surgery for an arachnoid cyst in the posterior fossa
− 3 y	Symptom onset	Development of pain along the cervical and thoracic spine
0	Presentation at the neurosurgical department	The patient seeks medical attention, and an MRI is performed. The patient is referred to the neurosurgical department for progressive neuropathic pain and the finding of multiple SACs in the cervical and thoracic spine, as well as intracranially in the posterior fossa and at the CCJ
4 m	1st operation at Th3–6	Several small cysts were successfully excised, and the spinal cord was decompressed without any complications
6 m	Clinical FU after 1st operation	Improved neurological status with total resolution of the upper thoracic pain. However, unchanged neck, arm, and lower thoracic pains. Histological analysis confirms the diagnosis of arachnoid cyst
7 m	MRI FU after 1st operation	Total decompression of the spinal cord at the operated level. No change in number and size of the other cysts. Unchanged severe compression of the spinal cord at the lower thoracic level
7.5 m	2nd operation at Th8–10	Several small cysts are successfully excised, and the spinal cord decompressed without any complications
10 m	Clinical FU after 2nd operation	Total resolution of the neuropathic pain corresponding to the operated level. However, worsening of the neck and arm pain
11 m	MRI FU after 2nd operation	The spinal cord was decompressed and there were no cysts left in the thoracic region. No intramedullary T2 signal changes were seen. Two of the untreated cervical cysts had enlarged and two decreased in size
1.5 y	MRI FU	There was a change in neither the number or size of the cysts nor the degree of spinal cord compression
3 y	Clinical FU	Recurrence of back pain, with steady neck and arm pain, which were managed conservatively
3 y	MRI FU	Pronounced progression of cysts which now extend intradurally throughout the whole cervical and thoracic spine. Unchanged status of the intracranial and CCJ cysts
7 y	MRI FU	Further increase in the size of several cysts and de novo cyst formation. Increased compression of the spinal cord. No intramedullary signal changes. Unchanged status of the intracranial and CCJ cysts
10 y	Clinical FU	Worsening pain managed with physiotherapy and different pharmacological regimens. Only partial pain relief on current medications. Renewed surgery deemed not meaningful due to the rapid recurrence and progression of the cysts

*FU*, follow-up; *CCJ*, craniocervical junction

### Admission and surgical management

**Fig. 1:**
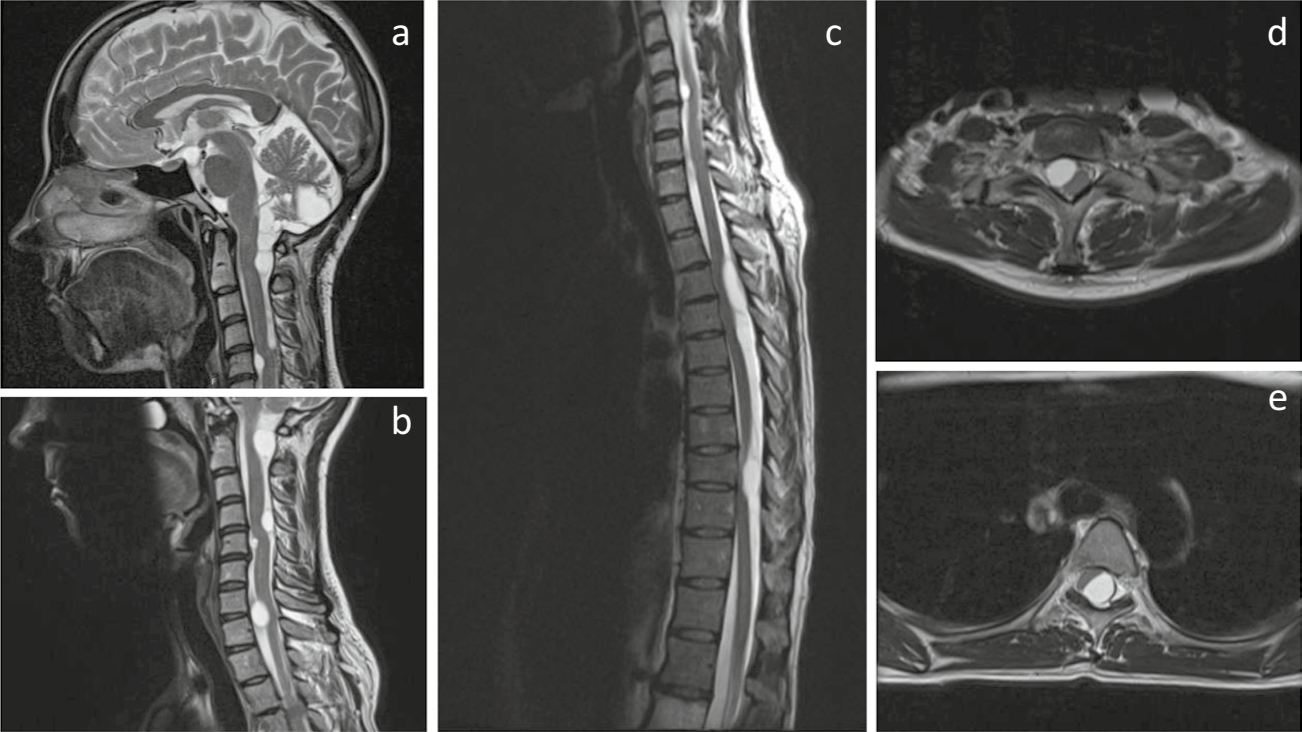
Sagittal MRI sections of the brain (**a**), cervical (**b**), and whole spine (**c**) as well as axial MRI at the C4 (**d**) and Th4 levels (**e**) on presentation at the neurosurgical department

The MRI revealed multiple intradural extramedullary cysts at different levels in the spinal canal, extending through both cervical and thoracic segments as well as intracranially in the posterior fossa and at the craniocervical junction (Fig. 1). At the level of Th3–Th6, a large, partially septated cyst was seen dorsal to the spinal cord and caused severe compression of the spinal cord especially at the level of Th4. At the Th9–Th10 level, multiple small cysts caused slight compression of the spinal cord. Other cysts in the cervical region were small and without significant spinal cord compression. The cysts appeared with high signal intensity on T2-weighted images equal to cerebrospinal fluid (CSF) but with slightly higher signal than expected for CSF on T1-weighted images. There was no diffusion restriction, and the cysts did not enhance after gadolinium contrast administration.

**Fig. 2:**
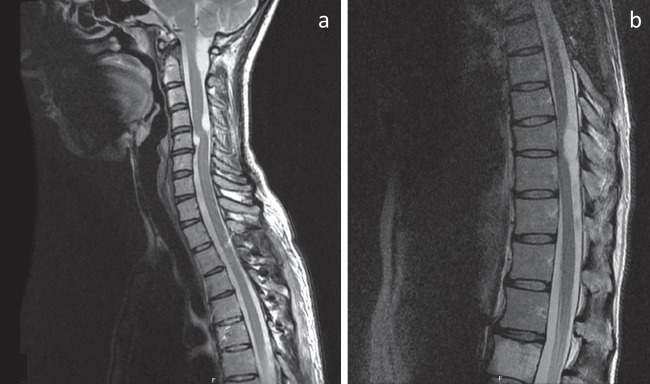
Sagittal MRI sections of the **a** cervical and upper thoracic and **b** lower thoracic spine after the initial spinal surgery performed at Th3–6

**Fig. 3:**
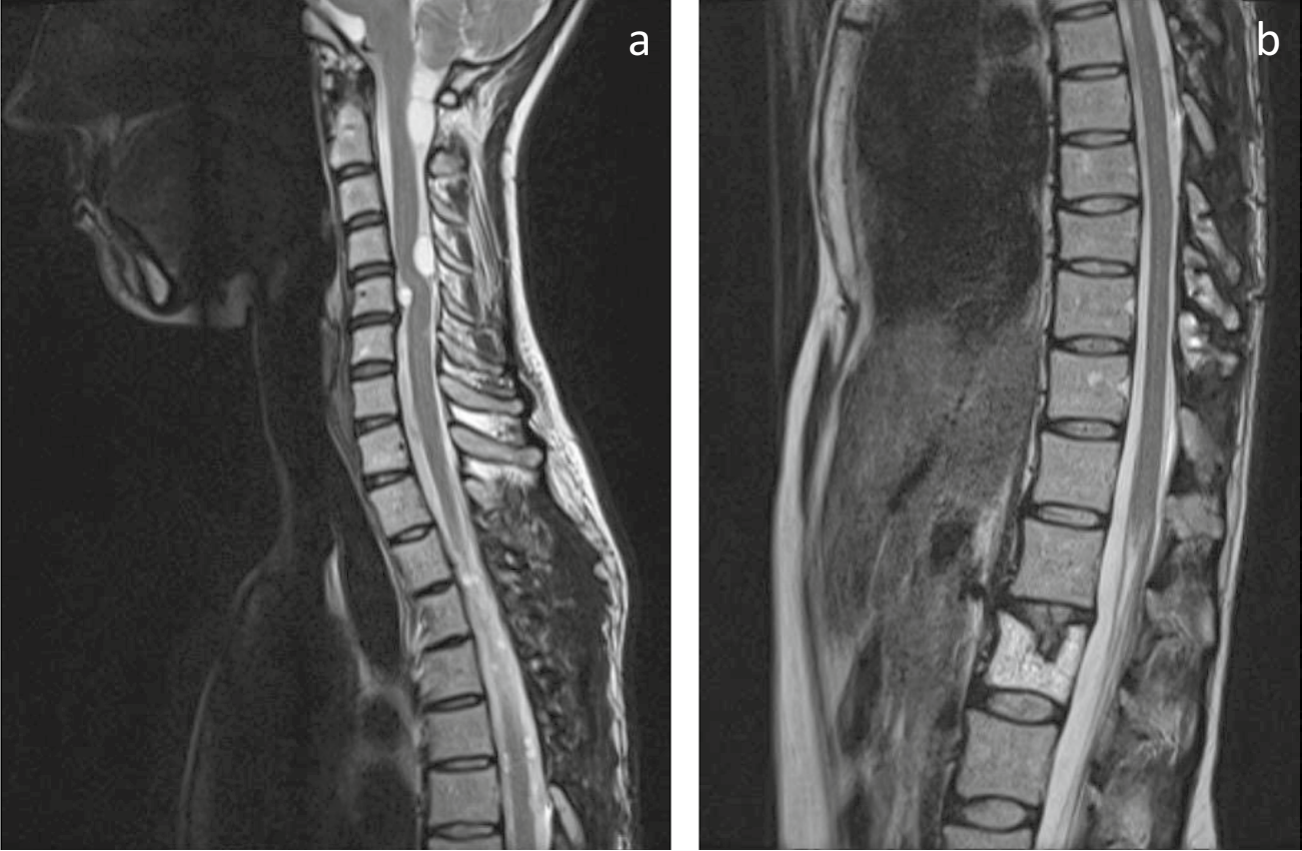
Sagittal MRI sections of the **a** cervical and upper thoracic and **b** lower thoracic spine after the second surgery performed at Th8–10

A stepwise surgical approach was adopted. In the first step, the most pronounced spinal cord compression was to be addressed. Depending on the outcome of this surgery, a second step to relieve less compressed areas was to be decided upon. The first surgery was performed between Th3 and Th6 where the cysts causing the most spinal cord compression were located. The spinous process of the vertebra adjacent to the cysts was identified using computed tomography guidance and marked with injection of a sterile carbon suspension. With the patient in the prone position, a posterior midline approach was performed. Laminectomy was conducted using an ultrasonic bone scalpel (Misonix Inc., Farmingdale, NY, USA). Under the microscope, the dura was incised and held open with sutures, allowing exposure of the cyst. The arachnoid was dissected sharply, and both cranial and caudal poles of the cyst were identified. The cysts were not adherent to the spinal cord and could be removed in their entirety. Watertight dura closure was achieved using resorbable sutures and fibrin sealant (Evicel, Ethicon, Raritan, NJ, USA) to support the repaired dura. The laminae were repositioned using microplates (CMF Medicon Surgical Inc., Jacksonville, Florida). Soft tissues were then sutured in layers to close the wound. The histological analysis confirmed the diagnosis of arachnoid cyst. The patient’s condition improved, and an MRI, 3 months after surgery, showed complete removal of the cysts at this level (Fig. 2). All other cysts remained unchanged. Consequently, a second surgery was scheduled to address the remaining symptoms. To allow sufficient recovery time, the second surgery, between Th8 and Th10, was performed 3 months later. A new MRI at 3 months after the second surgical procedure showed resolution of the cysts and the spinal cord compression (Fig. 3). The recovery period after each surgery was around 3 months, and as the patient’s symptoms had improved, the decision was made to follow the patient yearly with MRI and clinical follow-ups depending on symptoms.

### Follow-up

**Fig. 4:**
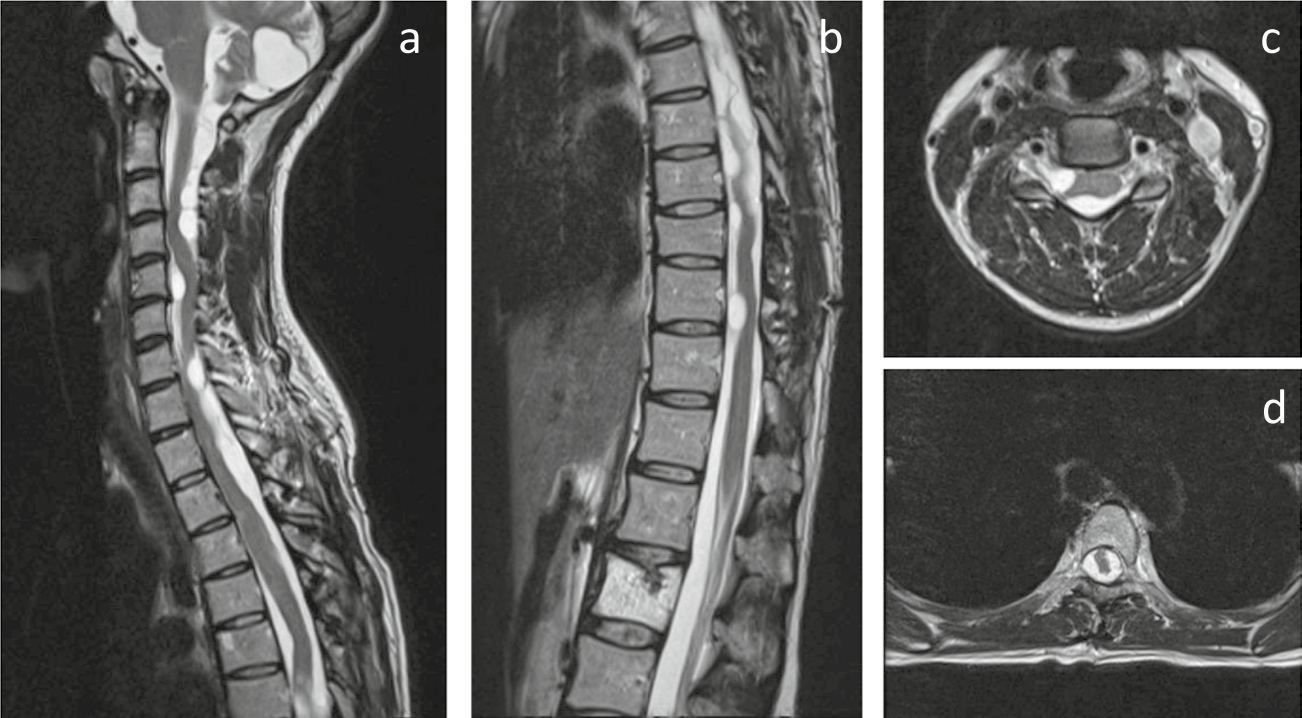
Sagittal MRI sections of the **a** cervical and upper thoracic and **b** the lower thoracic spine. Axial MRI sections at **c** C3 and **d** Th4, 3 years after presentation

Two years later, the patient developed radicular neuropathic pain of the upper extremities and lower lateral parts of the abdomen. A new MRI was performed in which a marked increase in the number and size of the cysts was found, extending almost continually from C2 to Th11. Spinal cord compression was present along most of this extension. An in-depth review of the current and previous MRIs by the Department of Neuroradiology resulted in the conclusion that there was a marked de novo cyst formation rather than only cyst recurrence. In contrast to the alarming MRI images, the symptoms were relatively mild, and since the cysts had recurred and developed despite two previous surgeries, it was decided to manage the patient conservatively with a trial of analgesic medications. A follow-up MRI was performed 6 months later, showing a stable condition (Fig. 4).

### Long-term follow-up

**Fig. 5:**
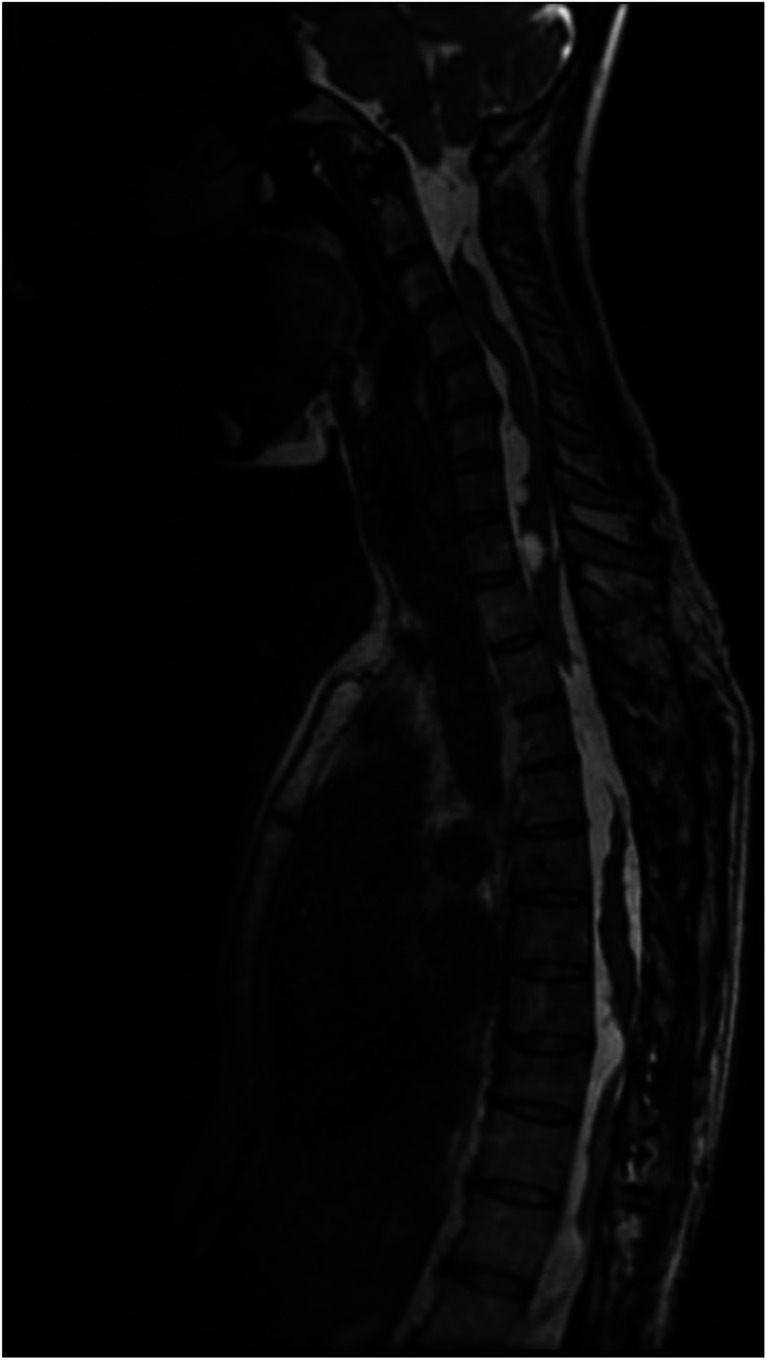
MRI of the whole spine at last follow-up (10 years after initial presentation)

Two years later, imaging again revealed a dramatic increase in the size and number of cysts. The cysts extended the entire length of the cervical and thoracic spine (Fig. 5). However, there was no obvious change in her symptoms, and the conservative strategy remained unchanged. At clinical follow-ups during the next 6 years, the patient gradually deteriorated in terms of pain and was managed at a pain clinic. Physiotherapy and several drug regimens, including acetaminophen, NSAIDs, chlorzoxazone, gabapentin, duloxetine, amitriptyline, and venlafaxine, were evaluated. At the latest follow-up, almost 10 years after the initial presentation, the pain still persisted but was partially managed with paracetamol (1 g × 4), gabapentin (300 mg × 1), amitriptyline (40 mg × 1), and celecoxib (200 mg × 2), allowing the patient to return to work part-time as a nurse with administrative duties.

## Discussion and conclusion

### Literature review

SACs, including the intradural and the extradural types, are rare causes of spinal cord compression [5]. Typically, these lesions are sporadic and single. A thorough review of the literature only revealed five cases of multiple and recurring intradural SACs (Table 2).

**Table 2:** Summary of the literature on cases of primary multiple spinal intradural arachnoid cysts

Study ID	Number of cases	Sex/age	Location of cysts	Association to genetic conditions	Management strategy	Recurrence and management	Main takeaways
Jamjoom 1991 [7]	1	M/32	L1–3, Th5–7, C6–Th4, C2–4, and Th2–3	Yes	Surgery	Yes, cystoperitoneal shunt	The presence of multiple arachnoid cysts should warrant further workup to investigate underlying genetical conditions
Osuka 1997 [9]	1	M/53	Multiple cysts between Th6 and Th12	No	Conservative	n/a	Both myelography and CT myelography were useful in establishing the diagnosis when MRI was inconclusive
Petridis 2009 [10]	1	F/44	Multiple cysts extending from T6 to L2	No	Surgery	n/a	Surgical treatment with partial cyst resection may provide immediate improvement of neurologic function
Zekaj 2016 [12]	1	F/47	Multiple cysts in the thoracic region	No	Surgery	No	Surgery should be offered early, as longstanding compression of the spinal cord may worsen the prognosis
Hayashi 2018 [6]	1	M/3	C2–5 and Th12–L2	No	Surgery	No	Despite the possibility of spontaneous disappearance of intradural SACs, surgery may be needed in case of severe symptoms
Present study	1	F/35	C1–2, C4–5, C7–Th1, Th3–6, and Th9–10	No	Surgery	Yes, conservative	Upon adequate indications, operative treatment should be offered to patients with multiple intradural SACs. Otherwise, adopting an expectant approach may be more appropriate

*M*, male; *F*, female; *C*, cervical; *Th*, thoracic; *L*, lumbar; *n/a*, not available

Jamjoom et al. reported on a 32-year-old male with five intradural SACs. The patient’s history was also remarkable for lymphedema, distichiasis, Arnold-Chiari malformation, and megaureters, suggestive of the diagnosis of lymphedema-distichiasis syndrome. In fact, cases of frequently recurring intradural SACs were reported in a family diagnosed with this condition [11]. The intradural SACs were found in the cervical, thoracic, and lumbar regions and warranted surgical management. Recurrence was reported for one of the cysts. It was managed with a cystoperitoneal shunt. Although some postoperative improvement was seen, the patient’s overall neurological function remained poor [7].

Osuka et al. reported on the difficulties in diagnosing a 53-year-old man with multiple thoracic intradural SACs using MRI. Conventional myelography and CT myelography were needed to identify the cyst. Due to the lack of pronounced symptoms, the patient declined the surgery and was instead followed in an outpatient setting [9].

Petridis et al. [10] reported on a 44-year-old female patient with severe pain and gait disturbance, diagnosed with multiple intradural SACs extending from Th6 to L2. Cyst puncture and evacuation of 20 ml CSF produced temporary relief for 2 months. Progression of lower limb weakness mandated surgery where partial cyst resection was performed in the areas with the most severe spinal cord compression. This resulted in an immediate improvement of the patient’s ambulatory function. However, her pain did not diminish. Long-term outcomes were not reported.

Zekaj et al. reported the formation of an intramedullary cyst after the removal of multiple thoracic intradural SACs in a 47-year-old female. The authors argue that the longstanding compression of the spinal cord caused by multiple cysts may have led to the formation of an intramedullary arachnoid cyst. In fact, the patient’s intradural SACs were conservatively managed during 3 years before surgical treatment. The authors suggested avoiding delay of treatment of such intradural SACs to prevent worsening prognosis as well as secondary complications such as intramedullary cysts [12].

Hayashi et al. described a 2-year-old with multiple spontaneously disappearing intradural arachnoid cysts [6]. The first cyst to appear was located in the subaxial cervical spine and resolved spontaneously together with its associated symptoms without any treatment during the initial hospital stay. Seven months later, the patient developed an intradural SAC at the thoracolumbar level. After 5 days, the symptoms resolved. Again, MRI revealed spontaneous resolution of the cyst before any attempt at treatment. One month later, similar symptoms recurred, and an MRI revealed a recurrence of the thoracolumbar cyst. Due to the severity of symptoms, the patient was surgically treated with complete cyst resection. No recurrence or residuals were detected on follow-up MRI [6].

### Summary

In the case presented, the patient developed several cysts extending intradurally from the posterior fossa to the lower thoracic levels. The etiology of the condition could not be defined. The patient denied any history of CNS trauma, bleeding, or infection and did not report any family history of similar symptoms or conditions. In the absence of these plausible etiologies, genetic factors were hypothesized to be mainly contributing to the condition. However, since the literature lacked reports of any genetic anomaly associated with multiple and recurring arachnoid cysts, the patient did not qualify for further genetic workups, as determined by the Department of Medical Genetics at our institution. Hence, based on these observations, the cysts were considered idiopathic.

In the reviewed literature, most patients with multiple intradural SACs were surgically treated. A conservative and expectant management was offered in one case [9]. In our case, surgical management was offered at the time of diagnosis. However, a more conservative approach was adopted after the initial surgeries due to the rapid recurrence of symptoms and progression in the size and number of the cysts. Similar to the case presented by Osuka et al., the argument was made that the symptoms were manageable, and the chances limited for long-term resolution of symptoms with surgery [9]. Nonetheless, frequent follow-ups with clinical assessments and MRI are essential to identify situations when the indications for surgery must be reassessed.

### Conclusion

In patients with symptoms of spinal cord and nerve root compression, the diagnosis of spinal arachnoid cysts may be considered. Multiple intradural SACs are rare, with only six cases being reported in the literature, including the one hereby presented. Most patients were offered surgical treatment at the time of diagnosis. However, cyst recurrence may be rapid, and it is important to consider conservative management strategies upon failure of other invasive approaches, especially in the absence of severe symptoms.

## Supplementary Information

Below is the link to the electronic supplementary material.

Supplementary file1 (PDF 1518 KB)

## Data Availability

The data may be provided upon reasonable request.
